# p53-guided response to nucleostemin loss in normal *versus* cancer cells

**DOI:** 10.1038/cddis.2015.377

**Published:** 2015-12-31

**Authors:** R Y L Tsai

**Affiliations:** 1 Center for Translational Cancer Research, Institute of Biosciences and Technology, Texas A&M University Health Science Center, Houston, TX, USA; 2 Department of Molecular and Cellular Medicine, Texas A&M University Health Science Center, College Station, TX, USA

Stem cells play pivotal roles in organogenesis, tissue regeneration, aging, and cancer formation. The two pillars that uphold the amazing power of stem cells in biology and, with hope, in medicine are their abilities to self-renew and conduct multi-lineage differentiation. One irreplaceable member in the self-renewal team is the mammalian nucleostemin (NS)^1^ (see a previous commentary for the need to distinguish between vertebrate and invertebrate NS with respect to their functionalities and hence mechanisms).^2^ Since its initial discovery as a neural stem cell-enriched gene more than a decade ago,^3^ NS has excited a great deal of interest. This excitement is largely driven by its translational potential, either as a diagnostic marker or as a therapeutic target. Over the years, many reports have indeed shown that the presence of NS is critical for the survival of proliferating cells and its expression is closely linked to tissue regeneration and late tumor progression. In contrast to its biological significance and utility, the mechanism of NS action in mammals has remained unsettled for years, until a recent discovery that reveals its role in protecting the integrity of the replicating genome by promoting homologous recombination (HR) repair of chromosomal and telomere damage.^4, 5, 6, 7^ This revelation sets mammalian NS free from the ideological constraints imposed by its nucleolar localization and GTP binding, and reconnects it with its root in self-renewal,^2^ as genome replication is accompanied by an increased risk of DNA damage that ultimately limits the proliferative life span of most dividing cells.

Earlier studies have indicated that mammalian NS contributes to p53 inactivation via physical interaction with MDM2.^8, 9^ Yet the need of p53 for NS to perform its obligatory function is disputed by multiple groups, who show that NS remains indispensable for p53-null cancer cells^2^ and that NS loss triggers DNA damage to the same extent in p53-wild type as in p53-null mouse embryonic fibroblast (MEF) cells.^5^ Despite that, those who decide to adopt this view would still need to explain why some outcomes of NS-deficient cells appear to be shaped in a p53-dependent manner. As the gatekeeper for the genome integrity, it is not surprising that p53 is still called upon to respond to the genotoxic damage caused by NS loss. Therefore, the route via which NS-deficient cells meet their final fates, be it cell cycle arrest, senescence, or apoptosis, should be influenced by their status of p53 or lack thereof.

In a recent study published in the journal of *Cell Death Discovery*, Huang *et al.*^10^ used two pairs of isogenic cell models to address the interplay between NS and p53 perturbation in MEF and human colorectal cancer (HCT116) cells. In MEF cells, NS depletion leads to G2 arrest regardless of their p53 status, but causes a marked increase of polyploid giant cells (PGCs) only in the absence of p53. The events leading to the G2 arrest in MEF cells include reprimo (RPRM) upregulation and sustained phosphorylation of cdc2, which appear in p53-wild type and p53-null cells, respectively and exclusively. On the other hand, the G2-arrest phenotype in cancer cells is underpinned by the upregulation of RPRM and the lack of dephosphorylation of phospho-cdc2 in both p53-wild type and null conditions. Based on the new information, the current model states that the essential function of NS resides in its genome-protective activity, whereas its direct MDM2-p53 regulatory function occurs mainly when its protein is released in bulk from the nucleolus during nucleolar stress. Although not directly linked to the fundamental activity of NS, p53 is still capable of orchestrating how NS-deficient cells respond to the G2/M arrest (Figure 1).

One of the responses to NS depletion that are notably different between normal and cancer cells is the growth disadvantage of p53 mutation in connection with PGC formation in normal cells. By comparison, the p53 status has relatively less impact on the growth-inhibitory response following NS depletion in cancer cells. PGC formation is a potential outcome of genotoxic insults, as are apoptosis and senescence. This endocycle event occurs when G2-arrested cells fail to enter or complete mitosis but instead reenter the G–S phase. Consequently, it is often seen in cells with a lax G2/M checkpoint control (e.g. p53 mutation) being exposed to genotoxic stress. Such is the case of p53-null and NS-depleted MEF cells. One biological advantage of forming PGCs is to preserve the genomic content by not forcing cells with incompletely replicated genome to divide and risk losing their chromosomes. Contrarily, Huang's study shows that PGC formation poses a major disadvantage to the population growth in MEF cells, suggesting that either PGC formation is an alternative way to rid of genomically defective cells or a longer experimental duration is needed to see its pro-survival effect.^11^ Compared with normal cells, the p53 status has a lesser impact not only on the growth of cancer cells in response to NS depletion but also on the molecular events leading to their G2/M arrest. Unlike MEF cells, where the increase of RPRM and the lack of dephosphorylation of phospho-cdc2 is dictated by their p53 status following NS depletion, these two events are increased in both p53-wild type and null cancer cells, even though RPRM is traditionally viewed as one of p53's targets. The lack of RPRM expression specifically in the PGC-enriched culture prompts the speculation that it might be involved in preventing G2-arrested cells from re-entering the endocycle. Overall, these findings indicate that p53-mutated cancer cells may find alternative ways to regain control of some effector events that are otherwise monitored by p53.

Finally, the use of NS as a therapeutic target requires finding agents that can specifically target this protein in its nucleolar hideout and avoiding the potential side effects on normal stem cells. The latter issue, other than lesion site-specific application, may be addressed systemically by exploiting the concept of synthetic lethality and tumor addiction. When embarking on the journey of taking NS out of the laboratory and into the clinics, those lesion-unique responses to NS deficiency may come in handy.

## Figures and Tables

**Figure 1:**
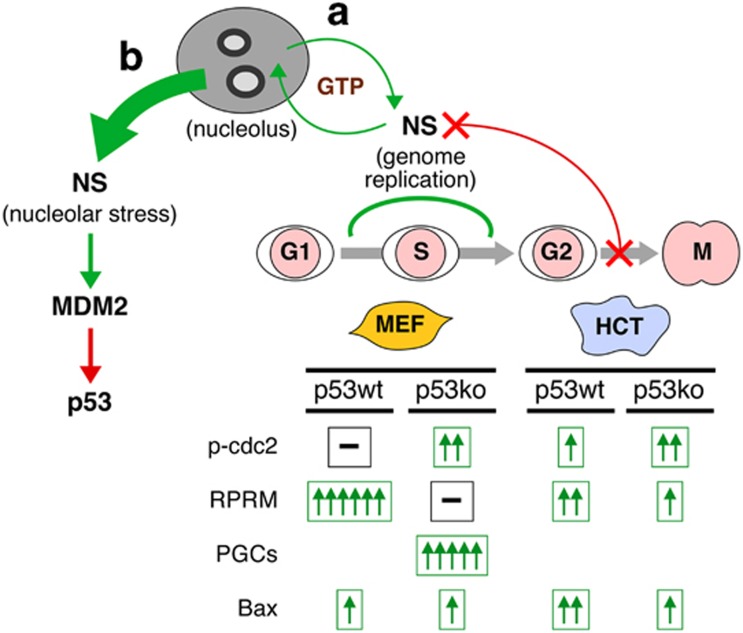
NS protects the integrity of the replicating genome to maintain self-renewal. (**a**) Our current knowledge supports an obligatory function of NS in maintaining the integrity of the replicating genome by promoting homologous recombination repair of DNA damage in the S-phase. This task is carried out by the nucleoplasmic NS, supplied from a nucleolar reservoir by a GTP-driven pump. Depletion of NS in proliferating cells results in G2 arrest. In mouse embryonic fibroblast (MEF) cells, NS deficiency triggers RPRM upregulation in the presence of p53 (p53wt) or sustained cdc2 phosphorylation and PGC formation in the absence of p53 (p53ko). In human colorectal tumor (HCT) cells, RPRM and phosphorylated cdc2 (p-cdc2) both increase with or without p53. (**b**) When the nucleolus is dissembled and spews out most of its contents in response to nucleolar stress, the abundant NS in the nucleoplasm will then interact with and stabilize MDM2. The green and red arrows indicate an excitatory/increase and inhibitory/decrease effect, respectively. The number of arrows denotes the degree of change
